# Reach and Acceptability of a Mobile Reminder Strategy and Facebook Group Intervention for Weight Management in Less Advantaged Adolescents: Insights From the PRALIMAP-INÈS Trial

**DOI:** 10.2196/mhealth.7657

**Published:** 2018-05-18

**Authors:** Laura Saez, Johanne Langlois, Karine Legrand, Marie-Hélène Quinet, Edith Lecomte, Abdou Y Omorou, Serge Briançon, PRALIMAP-INÈS Trial Group

**Affiliations:** ^1^ APEMAC EA4360 University of Lorraine Nancy France; ^2^ National Conservatory of Arts and Crafts Nancy France; ^3^ Clinical Epidemiology, Clinical Investigation Center National Institute for Health and Medical Research University Hospital Regional Center Nancy France; ^4^ Academy Rector of Nancy and Metz Nancy France; ^5^ APEMAC EA4360 University of Lorraine Metz France

**Keywords:** adolescent, social media, text messaging, overweight, socioeconomic factors, weight loss

## Abstract

**Background:**

Although information and communication technology interventions appear to be a promising means of reducing the health inequality gap in overweight and obesity prevention, research on information and communication technology interventions is lacking outside the Anglo-Saxon world.

**Objective:**

The aim of this study was to assess the reach and acceptability of 2 information and communication technology interventions delivered as part of a French nutritional program: an SMS text messaging (short message service, SMS) attendance-reminder for collective sessions strategy and a Facebook challenge group.

**Methods:**

This study sample comprised 262 socially less advantaged overweight adolescents aged between 13 and 18 years. The information and communication technology interventions were carried out during the 2013-2014 academic year in 33 French state-run schools. For the SMS attendance-reminder for collective sessions strategy, at the start of the academic year, adolescents were asked to give their mobile number. SMS attendance-reminders were sent shortly before each of the 5 collective sessions. For the Facebook challenge group, adolescents were invited to join a closed Facebook group in which challenges on physical activity and on diet were posted weekly. Process data and 2 sets of face-to-face interviews were also used to interpret participation rates and access to Facebook. Appreciation for both interventions was evaluated by a questionnaire at the end of the academic year.

**Results:**

Of the recruited adolescents, 79.0% (207/262) gave their mobile number, reflecting high access to a mobile phone. Giving a number was significantly more likely for girls (odds ratio [OR] 2.1, 95% CI 1.1-3.9; *P*=.02) and adolescents in a vocational or general high school as opposed to middle school (OR 1.0, 95% CI 0.4-2.7; OR 0.2, 95% CI 0.1-0.5; *P*<.001). Indicating a mobile number at the start of the year was not significantly associated with participation in collective sessions. Of the adolescents seen at the start-of-year face-to-face interviews, 78.1% (153/196) declared an interest in the Facebook challenge group, which implies having a Facebook account or being able to have access to one. However, only 21 adolescents went through the process of joining the group. Although there was satisfaction with the Facebook group among the participants, the low participation rate in the Facebook group does not allow conclusions to be drawn with confidence.

**Conclusions:**

The results are in line with the claim that using information and communication technologies in health programs is unlikely to widen health inequalities. However, in this population of French adolescents, mobile phone strategies seem more adapted to a high school context, and caution should be exercised with a younger audience. Although there is positive appreciation of the SMS attendance-reminders and a Facebook intervention is initially highly appealing to less advantaged adolescents, no evidence of impact could be demonstrated. These results highlight the difficulty in assessing the impact of specific interventions in complex health programs.

**Trial Registration:**

Clinicaltrials.gov NCT01688453; https://clinicaltrials.gov/ct2/show/NCT01688453 (Archived by WebCite at http://www.webcitation.org/6yy6EQ0SM)

## Introduction

### Background

Although there is evidence to suggest that the rates of obesity and overweight have stabilized in recent years, social disparities persist, both for adults and children, in developed countries [1,2]. Indeed, there is an inverse relationship between income category and the prevalence of obesity. The social gradient is clearly evidenced by all measures of social inequality: profession, level of education, family income, and even perception of wealth [1-5]. In France, the latest data show that the crude prevalence of obesity is 15%, but this figure rises to 24% for individuals belonging to the lowest income category [3], and social differences in obesity and overweight prevalence seem to be increasing not only for adults but also for children and adolescents [6,7].

It is, therefore, essential to develop health programs that do not widen health inequalities and tackle the different levels of social disadvantage, particularly in the field of obesity and overweight reduction. However, the features of such a program and the interventions it should encompass are as yet unclear and an urgent need for research on this topic has been expressed [8-10]. To fight against health inequalities, information and communication technologies (ICTs) are increasingly used in health programs, particularly social networking sites and mobile phones [11-13]. One of the main arguments for the use of ICTs in health programs is that it has the potential to engage with hard-to-reach populations [14-17]. Indeed, evidence is accumulating, particularly in developed countries, to suggest that ICTs transcend social class; young people especially seem to use social networks and mobile phones regardless of ethnicity, education or gender, and this appears to be true on a worldwide scale, including France [18-21].

More specifically, several studies have shown promising results of SMS text messaging (short message service, SMS) attendance-reminders especially in adolescent populations [22-26]. Adolescents are very receptive to the use of social media and especially Facebook [21,27-29]. Indeed, Facebook in particular was found to have the most widespread use relative to online support groups and blogging [18,30], as well as being the social networking site most used for research [11]. Furthermore, at a European level, Facebook is still being reported as the most used social networking site, with between 77% and 85% of adolescents aged between 13 and 16 years having a Facebook account [28]. It has also been shown that adolescents of low socioeconomic status (SES) may actually be more likely to have a Facebook account relative to those of higher SES [28]. A further argument for the use of Facebook is that several interventions using social media to reduce weight have found some evidence of a significant effect of social ties and post sharing, which are essential features of Facebook [31-35]. Although there is an increasing use of social media in health care contexts, recent reviews highlight several research gaps that still need to be filled including, for example, the need to report, precisely describe and evaluate such interventions but also specifically assessing the impact of social media in specific populations, such as minority groups [29,36-38].

### Objectives

The aim of this study was therefore to assess the reach and acceptability of an SMS attendance-reminder for collective sessions strategy and a Facebook challenge group for socially less advantaged adolescents, delivered in a health program addressing weight management in French adolescents.

## Methods

### Promotion de l’ALIMentation et l’Activité Physique-INEgalité de Santé (PRALIMAP-INÈS) Trial

This study was carried out during the 2013-2014 academic year within a larger research program running over a 3-year period for the prevention of overweight and obesity aimed at overweight and obese adolescents aged between 13 and 18 years attending grades 9 and 10 in 33 state-run middle-schools and high-schools in the Vosges department (north-eastern France). The full protocol of the PRALIMAP-INÈS (Promotion de l’ALIMentation et l’Activité Physique-INEgalité de Santé) trial is described elsewhere [39]. A letter was sent to adolescents’ home addresses, and they were included in the study unless their parents or legal guardian returned a refusal slip. Parents could return written consent, but this was not a requirement for inclusion and, not returning a refusal slip was considered as tacit consent leading to inclusion in the study. The trial was registered (Clinicaltrials.gov NCT01688453) and approved by the French consultative committee for treatment of information in health research (no. 12.299), the French National Commission for Data Protection and Liberties (no. 912372), and the French Persons Protection Committee (no. 2012/15). The core component of the PRALIMAP-INÈS program comprised 5 collective sessions offered throughout the school year. The adolescents were offered several other interventions, two of which were based on ICTs that are the focus of this study: an SMS attendance-reminder for the collective sessions and a Facebook challenge group. The PRALIMAP-INES program was multicomponent and designed so that all students could benefit from several, none, or all possible interventions, which were independent from each other. This study focuses on a subsample of the population enrolled in the main program consisting of 262 overweight and socially less advantaged adolescents, corresponding to the low and medium categories of the Family Affluence Scale (FAS) [40]. Recruitment strategies belong to the overall program and not specifically to the subpopulation.

#### Short Message Service Attendance-Reminder Strategy for Collective Sessions

The first ICT intervention was an SMS attendance-reminder strategy to encourage participants to attend the collective sessions organized in the schools. The 5 collective sessions were conducted in small groups and allowed participants to discuss themes related to healthy eating and physical activity. While filling in the questionnaires at the start of the academic year (T0), adolescents gave their mobile number or indicated that they did not have a mobile phone. For all participants, who gave their mobile phone number, SMS text messages were sent shortly before each collective session to remind them of the time and place of the session. For adolescents who did not give a mobile phone number, reminders were sent by email. Some school nurses took the initiative of providing further reminders to attend the collective sessions.

#### Facebook Challenge Group

The second ICT intervention, which was completely independent from the collective sessions and their associated SMS attendance-reminders, was a Facebook challenge group which was chosen among other possible interventions by a regional committee of student representatives and designed using The Reader-to-Leader Framework [41]. A test phase of the Facebook challenge group took place during the 2012-2013 academic year, at the end of which qualitative face-to-face interviews were carried out to assess appreciation and improve implementation for the 2013-2014 academic year. Changes following the test phase included: handing out a leaflet during the start-of-year screening process, using peer mediators to post the challenges after discussion with the coordinator, and adding a points system rewarding sharing of experience, peer support, and user-generated challenges. These strategies were aimed at increasing engagement, as this has been shown to be a key mediator of successful social media interventions [42]. Adolescents were invited to join a closed Facebook group in which 2 nutritional challenges, one on physical activity and one on diet, were posted on the group page on a weekly basis. As this intervention was only intended for the less advantaged subsample of the PRALIMAP-INÈS program, the Facebook group was closed so that only invited participants could join. To access the group, adolescents had to invite the group coordinator to be a Facebook friend. Indeed, some research has suggested that a private group may be acceptable and effective in certain contexts, as it may provide a judgment-free space facilitating goal achievement [43,44]. It was then possible to sign up for a challenge by clicking the *like* feature of Facebook. There were 2 types of challenges: those concerning physical activity, for example: “Twice a week, jump up and down 50 times. It’s even more fun using a jump-rope!,” and those concerning healthy eating such as: “For 1 month, cook a healthy meal for yourself and your family. Share your recipes by posting them on this page so everyone can enjoy!” The 2 peer mediators contacted the group coordinator with a challenge idea and following approval, posted it on the group page. See Multimedia Appendix 1 for an example screenshot.

### Data Collection

#### Reach

Access to the mobile phone intervention refers to indication of a mobile phone number when filling in the start-of-year questionnaires (T0). Access to Facebook is implied from having answered “yes” or “maybe” in terms of willingness to participate in the challenge group to the coach in the 2013-2014 start-of-year interviews (T0). Intention to participate was considered to indicate having a Facebook account or being able to have access to one. Participation was also considered an important indicator of reach. Adolescents were considered to have participated in the SMS-reminder strategy if they provided a mobile phone number. They were considered to have participated in the collective sessions if they were present for at least one of the sessions. The level of participation was also described using the number of attended collective sessions. Regarding the Facebook challenge group, adolescents were considered to have participated if they were a member of the group. Furthermore, process data was recorded to inform on participation in the individual nutritional challenges of the Facebook group. The following sociodemographic variables were also collected to describe the sample of participating students for both interventions: age, gender, FAS score, school type, boarding school status, family status, and type of parental consent given. Obesity status was determined by the obesity thresholds for age and gender according to the International Obesity Task Force [38].

#### Acceptability

Acceptability was evaluated using a mix of qualitative and quantitative indicators. Appreciation of SMS attendance-reminders was evaluated in the end-of-year questionnaire (T1) by asking adolescents whether they appreciated this mode of communication. Replying “completely agree” or “mostly agree” to the question asking adolescents whether they appreciated receiving SMS attendance-reminders for collective sessions were taken into account as positive appreciation.

For the Facebook group, appreciation and acceptability were collected in 2 waves of face-to-face qualitative interviews. The first was at the end of the test phase of the Facebook group in 2012-2013 academic year and the second at the start of the 2013-2014 academic year. Both were embedded in the PRALIMAP-INÈS general assessment: the 2012-2103 follow-up session and the 2013-2014 inclusion session. Both were organized through close collaboration between the school and research teams [39]. All eligible adolescents were, therefore, invited to participate in the interviews if they attended these sessions. The interviews consisted of a mix of closed and brief open-ended questions, and adolescents’ answers were noted directly during the interview. The interviews, at the end of the test phase, were carried out with 28 adolescents. Adolescents were asked to share their experience of the activity, particularly exploring barriers to participation. In the face-to-face interviews, with a coach at the start of the 2013-2104 academic year, adolescents were encouraged to give reasons explaining their interest or lack of interest in joining the Facebook group. Adolescent appreciation of the Facebook group was also evaluated in the end-of-year questionnaire (T1). Replying “completely agree” or “mostly agree” to any one of the following 3 propositions was considered to be positive appreciation: the challenges which were offered corresponded to what they expected, they found it useful to participate in the challenges, and they would recommend participating in the challenges to a friend.

### Analyses

Study sample characteristics, access to ICT, participation, and appreciation were described using percentages for categorical variables and mean (SD) for quantitative variables. Logistic regression models were used to identify sociodemographic factors, overweight status, and type of parental consent associated with access, participation, and appreciation. Bivariate analyses were used to assess independent associations. In bivariate analyses, a variable was made eligible for multivariate analyses if *P* value was ≤.02 in order to identify potential confounding factors [45]. For multivariate analyses, a stepwise selection method was used with a .05 level of entry and retention in the model. With each regression model, the odds ratio (OR) with 95% CI and *P* value were calculated. Statistical analyses were carried out using SAS 9.4 (SAS Inst, Cary, North Carolina, United States).

## Results

### Sample Characteristics

Sample characteristics are described in Table 1. The average age was 15.4 years and 56.5% (148/262) were girls. Of this sample of less advantaged adolescents, 24.4% (64/262) were obese, and 8.4% (22/262) were at the very low end of the FAS (having a FAS score of 1 or 2). In terms of school type, 44.7% (117/262) attended vocational high schools, 34.7% (91/262) general high schools, and 20.6% (54/262) middle schools. The vast majority lived with both their parents (76.3%, 200/262) and consent to participate in the program was mostly given tacitly (82.1%, 215/262). Out of the recruited adolescents, 77.9% (204/262) completed the end-of-year questionnaire. There was no difference in sociodemographic factors, overweight status, and type of parental consent between the adolescents who filled out an end-of-year questionnaire and those who did not. However, there was a significant difference between adolescents answering the specific questions on program appreciation and those who did not. The 115 adolescents answering were younger (*P*=.004) and more likely to have written, as opposed to tacit, consent from their parents with 31.3% (36/115) of those answering having written consent versus only 7.5% (11/146) of those not answering having written consent (*P*<.001).

### Access, Participation, and Appreciation

#### Flowchart

**Figure 1:**
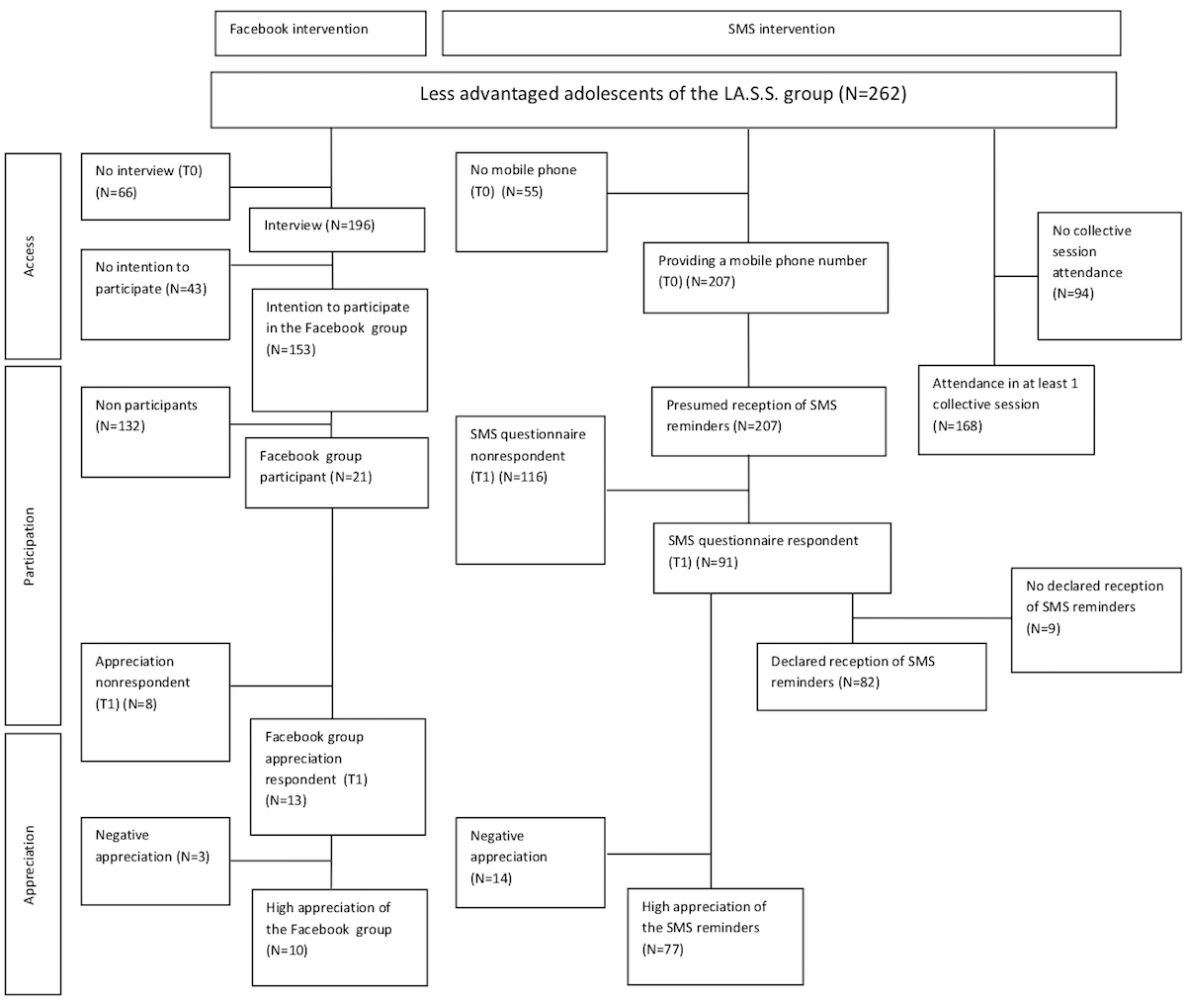
Flowchart describing rates of access, participation, and appreciation for the Facebook intervention and the intervention of short message service (SMS) attendance-reminders for collective sessions.

The flowchart describing the rates of access, participation, and appreciation for the Facebook intervention and the SMS attendance-reminder for collective sessions is presented in Figure 1.

#### Quantitative Data

Results describing access to a mobile phone and Facebook, as well as participation and appreciation of both the SMS attendance-reminder for collective sessions and the Facebook challenge group are presented in Table 2.

Of the adolescents considered in this study, 79.0% (207/262) provided their mobile phone number. Moreover, 25.2% (66/262) adolescents were not able to benefit from a start-of-year face-to-face interview with a coach. Of the adolescents that did attend the interview, 78.1% (153/196) declared an interest in participating in the Facebook group, which implies access to a Facebook account.

Only 8.0% (21/262) adolescents in the study sample participated in the Facebook group, and 64.1% (168/262) participated in at least one collective session. The average number of collective sessions attended was 3.4 of the 5 offered.

Of the adolescents who responded to the question on SMS attendance-reminder for collective sessions appreciation, 80.9% (93/115) had a positive appreciation of the SMS attendance-reminders. For the Facebook group, of the adolescents who responded to the questions on appreciation, 77% (10/13) had a positive appreciation of the Facebook group.

#### Qualitative Data

At the end of the test phase in 2012-2013 academic year, out of the 28 adolescents that were seen, 7 had participated in the Facebook group. For the participants, the main advantages of the group were that the challenges were motivating as they provided objectives, that the challenges gave ideas, and that they were perceived as fun. Several adolescents, however, mentioned lack of time to look at the challenges and not being able to spend time on Facebook at certain busy periods of the year. One adolescent girl also spontaneously mentioned: “there was not much point since I couldn’t do it with my friends.” Of the adolescents who did not participate, many reported not remembering being informed of the group, and several mentioned that the process to join the group seemed complicated so they did not take the time to look into it. However, 3 adolescents explained a general lack of interest in Facebook, and 3 others said they did not want to participate in any of the PRALIMAP-INÈS activities. None of the adolescents, who were interviewed, declared a lack of access to Facebook.

**Table 1:** Sociodemographic characteristics of the study sample (N=262).

Characteristics		Statistics
Age in years, mean (SD)		15.4 (0.8)
**Gender, n (%)**		
	Boys	114 (43.5)
	Girls	148 (56.5)
**Obesity status, n (%)**		
	No	198 (75.6)
	Yes	64 (24.4)
**FAS^a^ score, n (%)**		
	Very low FAS (1-2)	22 (8.4)
	Low FAS (3-4)	123 (46.9)
	Average FAS (5)	117 (44.7)
**School type, n (%)**		
	Vocational high school	117 (44.7)
	General high school	91 (34.7)
	Middle school	54 (20.6)
**School boarding status, n (%)**		
	Nonboarder	63 (24.4)
	Half-boarder	130 (50.4)
	Full boarder	65 (25.2)
**Family status, n (%)**		
	Two-parents	200 (76.3)
	Single parent	51 (19.5)
	Other	11 (4.2)
**Type of parental consent, n (%)**		
	Written parental consent	47 (17.9)
	Tacit parental consent	215 (82.1)

^a^FAS: Family Affluence Scale.

At the start of the 2013-2014 academic year (T0), of the 196 students benefiting from a face-to-face interview with a coach, 43 adolescents declared a lack of interest in the Facebook group. Only 17 adolescents mentioned a lack of access to Facebook. Other reasons for declaring not being interested in the Facebook challenge group and concerning 6 adolescents each were: not wanting to participate in any of the PRALIMAP-INÈS activities and not liking Facebook in general. The overwhelming reason for declaring being interested in the Facebook challenge group was to benefit from the rewards that could potentially be obtained through participation. Furthermore, 4 adolescents particularly mentioned liking Facebook in general and being happy with any intervention on this platform.

### Factors Associated With Access, Participation, and Appreciation

Significant results from the multivariate regression models for access, participation, and appreciation of the Facebook intervention and the SMS attendance-reminder strategy are presented in Table 3.

In terms of access to ICT, gender and school type were significantly associated with mobile phone access. Girls provided their mobile phone number more often than boys (84.5%, 125/148 vs 71.9%, 82/114; OR 2.1, 95% CI 1.1-3.9; *P*=.02). Over 80% of the adolescents in high school provided a mobile phone (85.5%, 100/117) in vocational high schools and 82.4% (75/91) in general high schools), but this was only the case for 59.3% (32/54) of adolescents in middle schools. This difference in access by school type was highly significant (*P*<.001; middle school OR 0.2, 95% CI 0.1-0.5, reference vocational high school).

No significant association was found for overweight status, SES, family status, and school boarding status. No variables were significantly associated with declared interest in joining the Facebook group and hence access to a Facebook account. However, it is important to note that adolescents providing a mobile phone were also more likely to have access to Facebook than those not having a mobile phone (81.3%, 126/155 vs 65.9% 27/41; χ^2^_1_=4.5; OR 2.2, 95% CI 1.1-4.8, *P*=.03).

Participation in at least one of the collective sessions was neither associated with indication of a mobile phone number at the beginning of the year nor access to a Facebook account. Furthermore, participation in the Facebook group was not associated with any demographic variables or participation in the collective sessions.

None of the demographic factors were associated with appreciation of the SMS attendance-reminders for collective sessions or the Facebook group, although there was a tendency toward a greater appreciation of receiving SMS reminders for older adolescents (OR 2.2; 95% CI 0.9-5.4; *P*=.06).

**Table 2:** Access, participation and appreciation of information and communication technology (ICT) interventions (N=262).

Access, participation, and appreciation of interventions			Statistics, n (%)
**Access**			
	Declared interest in Facebook group^a^ (n=196)		153 (78.1)
	Providing a mobile number at the start of the academic year		207 (79.0)
**Participation**			
	In at least 1 collective session		168 (64.1)
	In the Facebook group		21 (8.0)
	Declared reception of SMS^b^ attendance-reminders for collective sessions (all adolescents; n=116)		93 (80.2)
	Declared reception of SMS attendance-reminders for collective sessions (of adolescents concerned^c^; n=91)		82 (90.1)
**Appreciation**			
	Positive appreciation of the Facebook group (n=13)		10 (76.9)
	Positive appreciation of SMS attendance-reminders for collective sessions (all adolescents; n=115)		93 (80.9)
	Positive appreciation of SMS attendance-reminders for collective sessions (of adolescents concerned^c^; n=91)		77 (84.6)

^a^Among adolescents seen by the coach in the 2013-2014 start-of-year interviews (n=262 minus number missing for the item).

^b^SMS: short message service.

^c^Among adolescents both answering the questionnaire and having given a mobile phone number (n=207 minus number missing for the item).

**Table 3:** Multivariate regression models for access to a mobile phone and participation in collective sessions.

Characteristics		Short message service attendance-reminders							
		Mobile phone access				Collective session attendance			
		N	n (%)	Multivariate regression^a^		N	n (%)	Multivariate regression^a^	
				OR (95% CI)	*P* value^b^			OR (95% CI)	*P* value^b^
								
Age^c^ (OR for 1 year age increase)		262	207 (79.0)			262	168 (64.1)	0.7 (0.5-1)	.04
**Gender**					**.02**				
	Boys	114	82 (71.9)	1 Reference value		114	68 (59.6)		
	Girls	148	125 (84.5)	2.1 (1.1-3.9)		148	100 (67.6)		
**School typ**e					**<.001**				
	Professional high school	117	100 (85.5)	1 Reference value		117	76 (65.0)		
	General high school	91	75 (82.4)	0.8 (0.4-1.6)		91	53 (58.2)		
	Middle school	54	32 (59.3)	0.2 (0.1-0.5)		54	39 (72.2)		
**Parental consent**									**<.001**
	Written consent	47	35 (74.5)			47	45 (95.7)	1	
	Tacit consent	215	172 (80.0)			215	123 (57.2)	0.1 (0-0.3)	

^a^Only factors with a significant association at .2 alpha risk in the bivariate regressions were candidates for entering into the multivariate model (n=89).

^b^*P* value is the level of significance of the test of the OR against 1.

^c^For age, N, n, and % describe the total sample and as age is a quantitative variable, the OR corresponds to 1 year age increase.

## Discussion

### Principal Findings

A high level of ownership of mobile phones was found in adolescents of lower SES and was greater among girls and high school students. In this sample, sending SMS attendance-reminders for collective sessions had no association with attendance at face-to-face sessions. Facebook seems largely accessible across school type and gender and adolescents manifest widespread interest in a health intervention using this platform. However, the uptake of the Facebook intervention was very low.

### Strengths and Limitations

Outside of the Anglo-Saxon world, research on ICT interventions is severely lacking in the scientific literature. This paper endeavors to address this issue by presenting results on the development and implementation of 2 ICT interventions in France. A main strength of this study is the all-inclusive nature of the intervention. All state-run high schools in the Vosges department, comprising just under 400,000 inhabitants [46], participated in the study along with some middle schools who committed to the project following a special request by the PRALIMAP-INÈS steering committee. All adolescents attending these schools in grades 9 or 10 were weighed and measured and, if concerned, invited to attend the program. Another strength of this study is its systematic measurement of SES, which made it possible to focus particularly on adolescents from less advantaged backgrounds. Limitations of this study include the indirect measurement of access: not providing a mobile phone number was taken to mean not having one, and willingness to participate in the Facebook challenge group was considered to imply access to Facebook. Another limitation may be that over half of the adolescents included in the study did not answer the specific questions in the end-of-year questionnaire under study. However, the adolescents who did complete these questions only differed in that they were younger and more likely to have written, as opposed to tacit, consent from their parents which may suggest obtaining support from parents may improve response rates, although this is particularly difficult with older adolescents. Finally, the low participation in the Facebook challenge group can also be considered a limitation, as no statistical conclusions can be drawn with confidence.

### Using Mobile Phones and Facebook in Health Programs

Access to a mobile phone seemed to be very widespread across this sample of less advantaged adolescents and this was likely to be an underestimation since some adolescents may have deliberately chosen not to provide their mobile phone numbers. Adolescent girls seemed more likely to provide their phone numbers than boys, which may reflect greater distrust by boys for sharing their number in a health program. In fact, it was generally observed that girls had a higher participation rate in the PRALIMAP-INÈS program as a whole. Another explanation could be that girls, possibly more communicative, are actually more likely to own a mobile phone and this has been reported in French and Swiss reports [21,47], although these gender differences have been not found in Europe [48]. These findings suggest more research needs to be carried out in order to confirm a potential gender bias in mobile phone ownership or use in France. The result of more mobile phone numbers being provided in high schools as compared with middle schools reflects the well-established increase in mobile phone ownership with age, even if the age of first phone ownership is steadily decreasing [21,28,47]; and it has been stressed that it is frequent for adolescents to receive a mobile phone at certain life stages such as changing to a more senior school [28]. This seems to be particularly the case between middle school and high school in France [47]. These findings support the use of mobile phones as a strategy which is unlikely to widen the inequality gap when used at high school level, but they also raise the question of use of mobile phones at middle school, at least for less advantaged adolescents. The results of this study also support the claim that Facebook can reach all audiences, regardless of SES [14,27]. The fact that very few adolescents in the face-to-face interviews stated that they did not have a Facebook account further supports this claim. Interestingly, unlike mobile phones, there was no association between age or gender and access to Facebook suggesting that in terms of ICTs, Facebook may be better suited to reaching boys and younger audiences than mobile phones.

In terms of acceptability, adolescents seem to appreciate receiving attendance-reminder SMSs, and this finding is in line with other qualitative studies which indicate adolescent satisfaction with this mode of communication [24,49,50]. The tendency toward a greater appreciation of receiving SMS attendance-reminders for older adolescents is a further argument for their appropriate use at high school level. Despite widespread reach and high levels of acceptability in this sample, provision of a mobile phone number at the beginning of the year was not associated with participation in at least one collective session. It is likely that this result is due to the fact that adolescents were encouraged to participate in the collective sessions with several channels of information, which varied among participating schools and included: information given at the end of each session for the next one, written notes distributed in class on the day of the sessions, oral reminders by the school nurses, and collecting students directly from the classroom. It is therefore not possible to isolate the SMS component from attendance, and further research is necessary to establish the impact of these reminders.

Perhaps the most surprising result is the gap between initial interest in the Facebook challenge group, which is higher than the reported interest in Facebook use in another recent study [51], and the low participation rate. The fact that the Facebook intervention was chosen among others by the regional committee of student representatives and the high reported interest rate suggest that Facebook is an appropriate medium for this sample of adolescents. However, increasing visibility following the test phase was evidently not sufficient to address the perceived complicated process of joining the group, as suggested in the interviews carried out at the end of the 2012-2013 academic year. Although clearly favorable to a Facebook health intervention, it is likely that adolescents do not naturally associate Facebook with health-related goals and this may have been a barrier to the process of joining the group. Beyond access to the group itself, a major challenge is the difficulty of obtaining interaction and initiative from the participants [52,53]. Despite various strategies to encourage active participation and sharing found to be effective in other contexts [50] such as the use of peer mediators, Facebook polls, or a points system which rewarded comments and user-generated challenges, adolescents did not take an active role in the Facebook group. In the interviews, participating adolescents mentioned that they used the group to set themselves personal targets and to get new ideas which confirms that the group was used on a personal level, and the collective component was not perceived. This is consistent with the finding that the main motivation to join the group was to obtain potential rewards and no social motivation was reported. Perhaps the number of participants was too few to create a dynamic environment with core members creating content [54]. However, a more likely reason is that the Facebook challenge group in this study was a *closed* group to allow cross-group comparisons among PRALIMAP-INÈS program participants, and this may have been contrary to the principle of social media as an attract-and-join space, in particular including existing friends in the intervention [35]. Indeed, it has been suggested that school-aged adolescents use Facebook in order to maintain and strengthen social ties primarily within their existing close-knit friendship networks rather than looking outwards to publicly expose their opinions or create new social ties [55-57]. It therefore seems important to tap into preexisting friendship networks to encourage mutual support and increase motivation rather than create an artificial group composed of health program participants. Given the rapidly evolving nature of the connected world, it may be worth considering other, more instantaneous and increasingly popular platforms such as Instagram, WhatsApp, or Snapchat [58]. However, more research is needed to ascertain whether these mobile apps are not likely to widen health inequalities, especially, if they require a smartphone with mobile internet connection.

### Conclusions

The results of this study are consistent with the claim that using ICTs in health programs is unlikely to widen health inequalities: access to mobile phones and Facebook is high, and the acceptability of both SMS attendance-reminders and a nutritional challenge Facebook group is evidenced by the positive appreciation given for both interventions. However, no evidence of impact of the SMS attendance-reminders or the Facebook challenge group could be demonstrated in th**is** study. These results highlight the difficulty in assessing the impact of specific interventions in multisite complex health programs, especially in school settings. In terms of recommendations for future health programs in France, using mobile phones seems particularly adapted in a high school context but caution should be exercised as a gender bias is possible. Taken together, the results of the Facebook group suggest that Facebook, as a social media platform, is appealing to French adolescents of low SES and may be particularly suited to reach younger adolescents and boys. However, key improvements are necessary to increase participation, in particular adding adolescents directly to the group and enabling adolescents to do the challenges with their own friends. Given the rapidly evolving nature of social media, it is also crucial to continue assessing interest in various social media platforms and usage by the target population before health interventions, especially as there may be differences according to SES.

## Appendix

Multimedia Appendix 1 Screenshot of one of the Facebook challenges.

## Abbreviations

FAS: Family Affluence Scale
ICT: information and communication technology
SES: socioeconomic status
SMS: short message service
