# Supplementary material for: Reach and Acceptability of a Mobile Reminder Strategy and Facebook Group Intervention for Weight Management in Less Advantaged Adolescents: Insights From the PRALIMAP-INÈS Trial
Source: JMIR Mhealth Uhealth. 2018 May 18;6(5):e110. doi: 10.2196/mhealth.7657 (PMC5984273; doi:10.2196/mhealth.7657)
Supplement: Multimedia Appendix 1 [file mhealth_v6i5e110_app1.pdf]

Multimedia Appendix 1 : Screenshot of one of the Facebook challenges

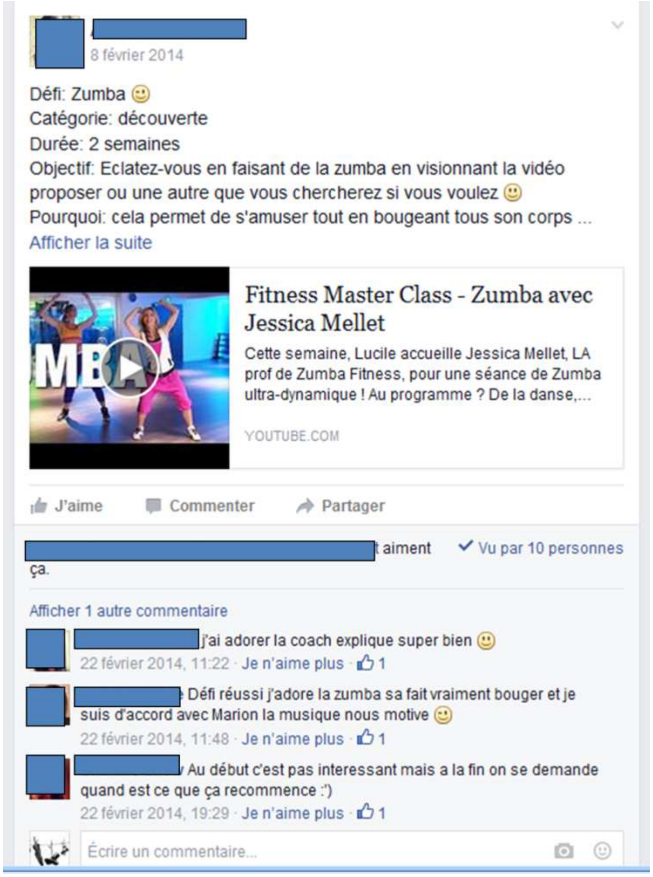

English Translation

Challenge : Zumba 🙂
Category : discovery
Length : two weeks
Objective : have fun by following this zumba video or any other you can find 🙂
Why : you can have fun and at the same time move your body…

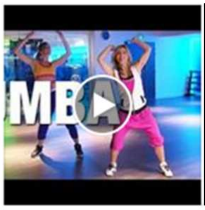

Fitness Master Class – Zumba with Jessica Mellet
This week, Lucile welcomes Jessica Mellet, LA Zumba Fitness instructor, for an ultra-dynamic Zumba session ! Programme of the day ? Danse…
YOUTUBE.COM

Comments

I loved it the coach explains things really well 🙂

I succeeded in this challenge I love zumba it really makes you move and I agree with X the music is really motivating 🙂

At the beginning I didn't fin dit interesting but by the end I wanted to start over again :')
